# Highlighting early detection of thyroid pathology and gestational diabetes effects on oxidative stress that provokes preterm delivery in thyroidology: Does that ring a bell?

**DOI:** 10.1016/j.clinsp.2023.100279

**Published:** 2023-09-30

**Authors:** Stefan Dugalic, Jovana Todorovic, Demet Sengul, Ilker Sengul, Eduardo Carvalho de Arruda Veiga, Jovana Plesinac, Milica Petronijevic, Maja Macura, Sonja Perkovic Kepeci, Miloš Milinčić, Andrija Pavlovic, Miroslava Gojnic

**Affiliations:** a Clinic for Obstetrics and Gynecology, University Clinical Centre of Serbia, Faculty of Medicine, University of Belgrade, Belgrade, Serbia; b Institute of Social Medicine, Faculty of Medicine, University of Belgrade, Belgrade, Serbia; c Department of Pathology, Giresun University Faculty of Medicine, Giresun, Turkey; d Division of Endocrine Surgery, Giresun University Faculty of Medicine, Giresun, Turkey; e Department of General Surgery, Giresun University Faculty of Medicine, Giresun, Turkey; f Department of Obstetrics and Gynecology, Universidade de São Paulo (FMRP-USP), Faculdade de Medicina de Ribeirão Preto, Hospital das Clínicas, São Paulo, SP, Brazil; g University of Belgrade, Faculty of Medicine, University Children Clinics, Belgrade, Serbia; h Faculty of Medicine, University of Belgrade, Belgrade, Serbia; i Clinic for Obstetrics and Gynecology, University Clinical Centre of Serbia, Belgrade, Serbia; j General Hospital Pancevo, Pancevo, Serbia

**Keywords:** Oxidative stress, Thyroid gland, Diabetes, Gestational, Thyroidology, Thyroidologists

## Abstract

**Objectives:**

*Ad fontes*, the status of the thyroid gland, and metabolic disturbance lead to the alteration of oxygenation. In pregnancy, it is particularly crucial to possess all predictive parameters.

**Methods:**

This cross-sectional study was conducted at the Clinic of Gynecology and Obstetrics, Clinical Center of Serbia, Belgrade, Serbia, between 2017 and 2021 which study included a total of 99 women who had been admitted for preterm delivery and had undergone thyroid analysis, detected Hashimoto thyroiditis, and Oral Glucose Tolerance Test (OGTT) 40 days after delivery and had pathological Homeostatic Model Assessment for Insulin Response (HOMA IR) indices. In the group of urgent patients with preterm delivery, we looked after not only routine Doppler of the umbilical artery, but we measured specific ratios such as the Cerebroplacental ratio (CP).

**Results:**

The mean maternal age was 32.23 ± 5.96 years and the mean gestational age was detected as 35.40 ± 2.39 weeks. The delivery was completed vaginally in 77 women (78%) and surgically in 22 (22%). The Mean APGAR score was 8.44 ± 1.18, the mean birth weight was 2666.87 ± 622.17g and the cases undergoing cesarean section had significantly higher values of pulsatility index (1.85 ± 0.27 vs. 1.34 ± 0.31) and CP (1.22 ± 0.26 vs. 0.47 ± 0.17).

**Conclusions:**

The introduction of Doppler sonography for blood flow assessment helps to form a complete clinical description of the patient, particularly in conditions where oxidative stress became provocative by the thyroid gland antibodies and gestational diabetes in Thyroidology.

## Highlights

• The status of the thyroid gland and metabolic disturbance leads to the alteration of oxygenation.
• The cases undergoing cesarean section had significantly higher values of the pulsatility index.
• The cases with cesarean section had significantly higher values of the cerebroplacental ratio.

## Introduction

Oxidative stress in pregnancy is provoked by the pathology of the thyroid gland and glucose metabolism. Ordinary Angel-Independent Doppler Indices have been developed for the analysis of blood flow through umbilical blood vessels in order to avoid observation bias. To this end, the most common indices in use have been the Resistance Index (RI), Pulsatility Index (PI), and Cerebroplacental ratio (CP).^1^ In most cases, those indices are measured in the Umbilical Artery (UA) and Medial Cerebral Artery (MCA) because they are the most available for measurements and are shown to be highly reproducible.^2^

The CP is calculated by using the pulsatility index, MCA, and UA. As such, the CP ratio is shown to be a more sensitive indicator of complications and poor pregnancy outcomes, compared to MCA and UA indices individually. In addition, a low CP ratio indicates blood redistribution toward cerebral circulation. Numerous studies all over the world have been purposed to determine reference values of CP for different gestational ages in different populations.^3^ Moreover, a negative correlation between RI, PI, and UA on one hand and gestational age between 18 and 40 weeks of gestation has been declared. The values of RI, PI, and MCA during pregnancy (18‒40 weeks of gestation) form parabola with a peak around 30 weeks of gestation which means that RI, PI, and MCA values will increase in the period of 18 to 30 gestational weeks, and then decrease until the end of pregnancy. Furthermore, the CP ratio also follows the parabola curve from 18 to 40 gestational weeks with a plateau from week 29 to week 31. It is suggested that these changes in hemodynamics are associated with the need for nutrients in the brain tissue in different gestational phases.^1^, ^2^, ^3^

It was previously hypothesized that the Doppler indices including PI, RI, and CP can be a marker of Gestational Diabetes Mellitus (GDM) and that changes in these indices might be associated with the disease development and progression^4^ which can indicate that the condition of the blood flow during pregnancy can reflect the pathophysiology of GDM. One of the hypotheses for this is that women with GDM, who have higher blood sugar levels, also have higher plasma viscosity, which is followed by higher resistance to the blood flow, lower flow speed, and abnormal blood perfusion, especially during early diastole.^5^ As such, the lower perfusion of the placenta can lead to poor or insufficient nutrient intake for the fetus, can negatively influence fetal development, delayed removal of the metabolites, and higher risk of fetal asphyxia.^4^ Of note, the use of Doppler indices can help establish the hemodynamic parameters, and timely diagnosis of abnormal perfusion and provide opportunities for the predictions of adverse pregnancy outcomes, having in mind that diabetes, including the GDM, is frequently associated with numerous, not only health but different psychological and social consequences.^6^

Combining the data from the Doppler indices with the gestational age, race, and ethnicity of pregnant women can allow clinicians to predict poor pregnancy outcomes.^7^ This article aimed to examine as least some of the parameters that can be associated with the possibility of adverse pregnancy outcomes.

## Material and methods

### Ethical aspects

The present study was conducted according to the declaration of Helsinki and approved by the Clinical Research and Ethics Committee linked to the School of Medicine University of Belgrade, under the 1322/IX-80 approval number.

### Study design

This cross-sectional study was conducted at the Clinic of Gynecology and Obstetrics, Clinical Center of Serbia, Belgrade, Serbia in the period of 2017‒2021. The study incorporated a total of 99 women who were admitted for preterm delivery and who had undergone analysis in Thyroidology. The values of anti-Peroxisomal (antiTPO), and Antithyroglobulin (antiTg) antibodies, were from OGTT 40 days after delivery and had pathological Homeostatic Model Assessment for Insulin Response (HOMA IR) indices. The data was gathered from the patient's records including the presence of an increased amount of amniotic fluid, accelerated fetal movements, disturbed circulation, Umbilical Cord Coding Index (UCCI), clinical and laboratory condition of the mother, BP (Blood Pressure) and CTG records (coded as normal and pathological). The cases for whom there were missing data were excluded from the study.

Preterm delivery was defined as the delivery before 37 weeks of gestation. HOMA IR is the score that is being used for the assessment of the presence of insulin resistance which utilizes the values of fasting blood glucose and fasting insulin using the formula: HOMA−IR(whenglucoseisinmmoL/L)=(fastingbloodglucosexfastinginsulin)/22.5.

Blood pressure was defined as in the pathological ranges above 140/90 mmHg^8^ and all the cases who were primiparas also suffered from hypertension during pregnancy, adequately regulated with small doses of antihypertensive medications. Blood pressure, several thrombocytes, creatinine, and transaminase levels had been recognized as normal in those on therapy. The Doppler indices were calculated using the ultrasound and the data on systolic peak (maximal velocity), which is the maximal velocity during the contraction of the fetal heart, the end-diastolic flow which represents the flow during the relaxation phase of the heartbeat, and mean velocity. The formulas used for calculations were as follows:

$$\text{RI}=\left(\text{Systolic}\;\text{velocity}-\;\text{Diastolic}\frac{\text{velocity}}{\text{Systolic}}\text{velocity}\right);\;\text{PI}=\left(\text{Systolic}\;\text{velocity}-\;\text{Diastolic}\frac{\text{velocity}}{\text{mean}}\text{velocity}\right).$$

The cerebroplacental ratio is calculated as the ratio between the pulsatility index of the MCA and the pulsatility index of the UA, as the use of PI was determined to be the preferred method in recent studies.^9^ The CPR was calculated as follows: $\text{CPR}=\text{PI}\frac{\text{mca}}{\text{PIua}}.$

### Statistical analysis

Statistical analyses, *per se*, were performed using the methods of descriptive and analytical statistics. The differences between the categorical variables were examined using the χ2 test. In addition, the differences between the numerical variables with normal distribution were examined using the student *t*-test. Furthermore, the differences between the numerical variables without the normal distribution were examined utilizing the Mann-Whitney *U* test. *In fine*, the normality was examined using the Kolmogorov-Smirnov Test. All analyses were done using the Statistical Package for Social Sciences SPSS 22.0.

## Results

All the women included in the study were primiparas and the mean maternal age was 32.23 ± 5.96 years and the mean gestational age was 35.40 ± 2.39 weeks. The mean BMI of pregnant women was 28.70 ± 7.35 kg/m^2^ and the delivery had been completed vaginally in 77 women (78%) and surgically in 22 women (22%). The Mean Apgar score was 8.44 ± 1.18, and the mean birth weight was 2666.87 ± 622.17g (Table 1). Poor UCCI, pathological BP, and poor redistribution had been more frequent in patients undergoing cesarean section (63.6% vs. 4.1%, 100% vs. 0%, 49.9% vs. 0%, and 95.5% vs. 1.4%, respectively) compared to patients with vaginal delivery (Table 2). The cases undergoing cesarean section had significantly higher values of PI (1.85 ± 0.27 vs. 1.34 ± 0.31), PI Artery cerebri media (PI Acm) (2.19 ± 0.09 vs. 0.61 ± 0.24), and CPR (1.22 ± 0.26 vs. 0.47 ± 0.17) compared to patients with vaginal delivery (Table 3).

**Table 1:** Patient characteristics, mode of delivery, and offspring characteristics after delivery.

**Characteristics**	**(X** **± SD)**
Maternal age	32.23 ± 5.96
Maternal BMI	28.70 ± 7.35
Gestational age	35.40 ± 2.39
Mode of delivery	
Vaginal	77 (78%)
Cesarian section	22 (22%)
Apgar score	8.44 ± 1.18
Birth weight	2666.87 ± 622.17

**Table 2:** Differences in patients’ clinical characteristics depending on the delivery mode.

**Characteristics**	**Vaginal delivery, n (%)**	**Cesarean delivery, n (%)**	**p-value**
**UCCI**			
1	70 (95.9)	8 (36.4)	
2	3 (4.1)	14 (63.6)	< 0.001
**CTG**			
Without disease	72 (100)	0 (0)	
Pathological	0 (0)	22 (100)	< 0.001
**BP**			
Without disease	72 (100)	13 (59.1)	
Pathological	0 (0)	9 (49.9)	< 0.001
**Redistribution**			
1	73 (98.6)	1 (4.5)	
2	1 (1.4)	21 (95.5)	< 0.001

**Table 3:** Differences in total pulsatility index, pulsatility index in the middle cerebral artery, and the cerebroplacental ratio between groups.

**Characteristics**	**Vaginal delivery, n (%)**	**Cesarean delivery, n (%)**	**p-value**
PI (X ± SD)	1.34 ± 0.31	1.85 ± 0.27	< 0.001
PI MCA (X ± SD)	0.61 ± 0.24	2.19 ± 0.09	< 0.001
CPR (X ± SD)	0.47 ± 0.17	1.22 ± 0.26	< 0.001

## Discussion

Patients who delivered in the period of theoretical preterm deliveries had been analyzed in the present study. No ordinary reason for preterm delivery (excluding anemia, hematological disorders, infection, and progesterone deficiency) has been noticed, but after looking after the primary condition and saving mother and baby, we recognized that the thyroid pathology and gestational diabetes were only pathological elements that have made oxidative stress in uteroplacental and fetoplacental circulation.

The present study revealed the significant differences between pregnant who had a vaginal delivery and pregnant who had delivery by the Caesarean section in frequencies of pathological Cardiotocography (CTGs), high blood pressure, Umbilical Cord Coding Index (UCCI), and presence of blood redistribution. Of note, significant differences in the Doppler examination for en-suite measurements in two groups (RI, PI, and CPR) have been detected indicating the higher possibility of necessity for operative delivery among women who had higher values of Doppler indices and stressing their importance in the preparation of the Obstetricians for this mode of delivery.

Previously, observation of classic parameters of fetal capacity, BP, and CTG did not give a clear prediction of possible complications or explanation of existing circumstances.^10^^,^^11^ The introduction of regular following of blood flow impedance in UA and CMA provided evidence that in those pregnancies in which the elements of preterm labor exist, the CPR ratio goes with the redistribution, that is, the fetus provides adequate circulation within the central nervous system for itself, even in the period of early gestation which is a consequence of poor circulation in UA and difficulties in blood supply in a fetal organism.^12^ Previous studies have shown that oxidative stress during delivery and in the perinatal period may cause fetal complications, as well. One of those studies aimed to assess the role of oxidative stress in perinatal hypoxic-ischemic brain injury using the activity of Glutathione Peroxidase (GPX) in Cerebrospinal Fluid (CSF) as an indirect biomarker of free radical production in the hypoxic brain and its correlation with the level of Neuron-Specific Enolase (NSE)^13^ which is an enzyme and good biomarker of extended brain injury which happens due to hypoxia and ischemia in fetal brain tissue.^14^

A possible interpretation of preterm delivery is the so-called “fetal escape” from the environment where metabolic disorder disturbs further intrauterine fetal existence. This occurs in different endocrinological disorders.^15^ The phenomenon of stress – fetal adrenal awakening and trying in order to escape the “space” which is not adequate for further growth and development, could be an explanation for preterm delivery in these situations.^16^ The fetus tolerates hypoxemia as a consequence of disturbed metabolism, given the fact that attenuated concentration of blood oxygen represents the consequence of metabolic disturbance. That kind of disturbance leads to fetal adrenal activation, known as the stress phenomenon, activating the early fetal maturation axis.^17^ The lower nutrient intake in the fetuses of pregnant women with the pathological values of Doppler indices may be associated with a higher likelihood of preterm delivery.^18^ As the prevalence of the various risk factors for GDM, among which is polycystic ovarian syndrome, is rising worldwide^19^, there is necessary to anticipate the possible complications among these patients. The authors have not noticed any other morbidity risk as family malignant potential^20^, ^21^, ^22^ or rather a frequent pathology in women as endometriosis.^23^, ^24^, ^25^

Delivery is a stress phenomenon itself, and, repeated hypoxic events in the fetus. A fetus without a compensatory capacity for adequate vaginal delivery had been delivered surgically. Those fetuses who had been delivered surgically had adequate APGAR scores, although their circulation at the admission to the hospital was beyond the level of compensatory effect and entered at the beginning of decompensation.

## Conclusion

The importance of early detection of the status of the thyroid gland in Thyroidology and metabolic disturbance changes the oxygenation. Introduction of Doppler sonography for blood flow assessment in UA and MCA, as well as CP index, along with UCCI is part which helps to form a complete clinical description of the patient, along with BP and CTG, especially in conditions like glucose metabolism disorder not detected in a timely manner that is the way to decrease the morbidity and mortality of neonates, get better APGAR scores, and attenuate the number of surgical deliveries. The authors postulate that the so-called analysis of thyroid-stimulating hormone, free fractions of thyroid hormones, and antibodies, in Thyroidology, as well as oral glucose tests, should be performed in all pregnancies, even physiological status. *Bene diagnoscitur, bene curatur.*

## Authors' contributions

Conceptualization, SD, JT, MG; Data curation, MG, SD, JP, MP, MM, SPK; Formal analysis, JT; Investigation, SD, DS, IS, ECAV, AP, JP, MP, MM, SPK, MJ; Methodology MG, SD, DS, IS; Software, AP; Project administration, SD; Resources, SD, JP, MP, SPK, AP; Validation, SD, JT, DS, IS, ECAV, AP, JP, MP, MM, SPK, AP, MG; Visualization, SD, JT, DS, IS, ECAV, AP, JP, MP, MM, SPK, AP, MG; Writing-original draft preparation, SD, JT, DS, IS, JP, MP, SPK, MM, AP, MG; Writing-review and editing, DS, IS, SD, JT, ECAV, AP, JP, MP, MM, SPK, MG; Supervision, DS, IS, SD, JT, MG. All authors have agreed to whole the manuscript before publishing.

## Declaration of Competing Interest

The authors declare no conflicts of interest.
